# The Motivations for and Well-Being Implications of Social Media Use at Work among Millennials and Members of Former Generations

**DOI:** 10.3390/ijerph18020803

**Published:** 2021-01-19

**Authors:** Reetta Oksa, Tiina Saari, Markus Kaakinen, Atte Oksanen

**Affiliations:** 1 Faculty of Social Sciences, Tampere University, 33100 Tampere, Finland; tiina.saari@tuni.fi (T.S.); atte.oksanen@tuni.fi (A.O.); 2 Institute of Criminology and Legal Policy, University of Helsinki, 00100 Helsinki, Finland; markus.kaakinen@helsinki.fi

**Keywords:** social media, work life, millennials, technostress, burnout, psychological distress

## Abstract

Working life has digitalized considerably in recent decades and organizations have taken into use new forms of collaborative technologies such as social media platforms. This study examined the relationship between social media use at work and well-being at work for millennials and members of former generations in Finland. The research data contained focus group interviews (*N* = 52), an expert organization survey (*N* = 563), and a nationally representative survey (*N* = 1817). Well-being measures included technostress, burnout, psychological distress, and a set of background variables. Content analysis and linear regression models were used as analysis methods. The results showed that millennials have various intrinsic and extrinsic motivations for social media use at work. Intrinsic motivations included employees’ personal choice and their pure interest to follow the market and discussions in their own field. Extrinsic motivations were related mainly to organizations’ work culture and personal branding. The survey findings revealed, however, that millennials were not only more active social media users for work, but they also experienced higher technostress and burnout than members of former generations. Social media use motivations were associated with both higher and lower technostress and burnout depending on motivation, indicating that social media use can have both positive and negative effects. Overall, our findings suggest that employees tend to utilize social media more if their needs for autonomy, competence, and relatedness are fulfilled.

## 1. Introduction

Working life has digitized considerably in recent decades and the progress still goes on [1]. Technology Acceptance Model (TAM) is widely used to explain user intentions [2] and intrinsic and extrinsic motivations to usage have established already in the early stages [3]. In general, younger employees tend to have a more positive attitude toward technology [4] (Morris & Venkatesh, 2000) and adapt more easily to new technologies compared to their older colleagues [5,6]. Younger employees lay their usage more on attitudinal base and older employees on social and process factors [4]. However, personality plays even greater role than age [7]. Technology acceptance has also been associated with work engagement, which highlights its importance for employee well-being [8].

Within the last decade, organizations have implemented more advanced forms of professional technology such as enterprise social media platforms [9,10,11]. However, younger employees may be more skeptical about the usefulness of social media for work compared to older employees [12]. Social media use at work is defined in this article as the use of internal corporate platforms such as Microsoft Teams or public social media platforms such as LinkedIn, through which employees use in their current workplace to create and maintain useful social networks [10,13], and to follow, share, and produce work or related content internally or to public audiences [10]. Although some studies exist, more research is needed on motivations to use social media for work purposes especially among different generations and the related well-being implications.

The work life is getting more technology intensive and technostress, in other words, technology related stress that employees find challenging to cope with, is also a pervasive issue in organizations [14,15,16,17], which has been further provoked by the COVID-19 [18]. Remarkably, younger employees are associated with higher levels of information technology (IT) related strain [19] and technostress compared to their older colleagues [20]. Technology can also stimulate burnout [21]. Nevertheless, the positive consequences of digitalization exist, and nearly a fifth of Finnish employees feel that it has decreased the strain and over half think that it has increased the work productivity and transparency [22]. Pirkkalainen and colleagues also pointed out that normative pressure and information load enhance IT engagement, which is higher for younger employees [19]. IT engagement can also foster IT enabled work productivity [19].

Members of Generation Y or millennials who were born in the 1980s and 1990s, are also known as digital natives [6] and the Net Generation [23]. They grew up in a digitized world and have had the opportunity to use and participate in various Internet based services and communities from their earliest stages [24,25]. Although millennials are technologically savvy and play active roles in work life, the workforce is growing steadily older in Europe, and the number of employees over 50 years old (31%) has surpassed the number of employees under 35 years old (30%) [26]. Therefore, organizations need to consider that employees can have different sets of technical experience and skills and the underlying motives to use technology can also vary. Therefore, the current study examines the relationship between social media use at work and well-being at work for millennials and members of former generations in Finland. In this study, our first aim is to explore millennials’ motivations and methods of professional social media use by examining qualitative data. We then analyze quantitative data of five expert organizations from finance, telecommunications, personnel services, publishing, and retail occupational fields and a nationally representative sample of Finnish employees to discern employees’ motivations for social media use at work and relationship to technostress, burnout, and psychological distress.

### 1.1. Digitalization and Well-Being in Contemporary Work Life

Finland has a long history in technological excellence with companies such as Nokia and is a leader in digitalization [27]. In Finland, 24% of employees’ work is ICT-enabled and can be completed regardless of their location [28]. In year 2018, a vast majority (91%) of employees in Finland used IT in their work [22], but only a third used social media for work purposes [29]. Nevertheless, social media use has increased steadily in organizations in recent years [30,31,32]. In 2018, the main purposes of social media use of Finnish employees were knowledge sharing (86%), information retrieval (83%), networking and collaboration (73%), customer service (53%), sales and marketing (43%), and product and service development (38%). Moreover, employees aged under 25 (32%) and employees aged 35–44 (33%) used social media at work most actively, although the difference was not considerable compared to employees aged 45–54 (30%) [29].

Social media provides numerous advantages for organizations. Internal social media platforms such as Microsoft Teams can improve organizational information and knowledge sharing and enhance internal communication practices [10,11,31]. Social media use can have an encouraging influence on collaboration and can enhance a sense of community across the company irrespective of physical location [30,33,34,35]. It can also have positive consequences for work performance and productivity [34,36]. Employees also utilize public social media platforms such as Twitter for professional development purposes, networking, and stakeholder management [30,37]. Moreover, organizations use these external platforms for marketing and branding, which enables them to reach diverse client audiences easily [9,38].

However, new forms of technology use can jeopardize employees’ well-being and induce psychosocial risks such as communication problems and leadership challenges in the creation of affective and cognitive processes for teams [39]. Moreover, the formation of in-groups, discrimination [10], and workplace cyberbullying [40] are becoming more common. As social media applications are nearly ubiquitous, they can erode boundaries between people’s private and professional lives because employees can access their work anywhere and at any time. Thus, work can easily spill over to free time [41] and challenge individuals to manage their work time and workload [8] and can have negative influence on employee productivity and organizational effectiveness in general [42]. Social media can increase communication, information, and social overload [32]. In particular, constant connectivity is induced by social media push notifications and messages, which can distract people from their work and lead to concentration and sleep problems, exhaustion, burnout and technostress [41,43,44,45]. Employees experience technostress when technology use challenges their ability to cope with the technology related demands [46,47]. This can lead to negative consequences such as strain and reduced well-being [46,48]. Indeed, a third of Finnish employees stated that digital devices and applications have increased their strain at work [22].

Overall, contemporary work life is ever more demanding, which has severe consequences for organizations. A third of Finnish employees aged 18–35 consider their work mentally and physically straining [29]. In contrast, older employees’ ability to work has improved in terms of mental and psychical strains since 2002 [29]. Concentration and memory problems are more common among employees aged 25–45, and women aged 25–34 experience the most stress [22]. Stress is produced by stressors, which elicit the employee’s negative psychological response to the stressor (i.e., strain) [48]. Anxiety, fear, and depression are examples of stress consequences [49]. Moreover, burnout is a more serve consequence of diminished job resources due to high demands at work, such as time demands or work overload [50]. Burnout comprises three dimensions: exhaustion, cynicism, and reduced professional efficacy [51]. Burnout also predicts longer sick leaves from work [52]. Consequently, in recent years, stress and burnout have become significant problems in work life.

Although technical skills are almost a necessity in the modern work life, the motivations of use can vary from employers to highly encouraging employees to use social media in professional context to employees utilizing it from their free will [53,54,55]. To this point, the motivations for social media use at work have been studied mainly by utilizing TAM [56,57], gratifications theory [58,59], and affordance lenses [11,60]. Furthermore, studies are conducted from hedonistic and utilitarian perspectives [36,61] and by discovering intrinsic, extrinsic, and apathetic motivations for social media use at work [55,62]. However, studies on self-determination theory (SDT) in work-related social media context are still scarce [63,64,65].

SDT is a theory that demonstrates individuals’ psychological development and goal-oriented motivational behavior [66], which is differentiated by intrinsic motivation (doing something that genuinely interest) and extrinsic motivation (doing something on the grounds of certain outcome) [67]. SDT provides a good framework for understanding the more innate motivations behind social media use, taking into account users’ basic needs for competence, autonomy and relatedness, and the relationship to well-being in work context [68] (p. 4). The SDT theory encompasses three psychological needs people have: autonomy (i.e., a sense of volition), competency (i.e., the ability to use one’s skills and capabilities), and relatedness (i.e., a sense of social belonging), which are affected by a person’s social circumstances and individual differences [66]. Meeting these basic needs fosters intrinsic motivation, self-regulation, and mental well-being [69]. Intrinsically motivated people tend to become engrossed in tasks they genuinely enjoy rather than aiming to accomplish external outcomes or obtain rewards, which is more typical of extrinsically motivated people [66]. However, extrinsic motivation as has its place. There are four regulation types of extrinsic motivation: external, introjected, identification, and integration, which represents the most autonomous behavior with assimilated regulations although the behavior itself is done on grounds of some instrumental value [67]. Because intrinsic motivation fulfills basic psychological needs, it is typically associated with positive well-being consequences, whereas extrinsic motivation may have the opposite impact and lead to negative well-being consequences e.g., [70,71,72].

### 1.2. Generations from Baby Boomers to Millennials

The term *Generation* may refer to either a familial generation or a social generation. The latter is a cohort of people born within the same date range. However, this population forms a generation only in a statistical sense. Being part of a generation in a social sense also requires people to share similar sociocultural experiences [73,74,75]. Shared experiences can include fundamental changes such as industrialization, cataclysmic events, or tragedies such as war [74].

Members of the generation born after the Second World War are called baby boomers; this name refers to the generation’s massive size. Most sources identify baby boomers as people born between the early 1940s and the mid-1960s [76,77]. The baby boomers were followed by members of Generation X, who were born during the late 1960s and the 1970s [76,78]. Once again, there is no single time range for this generation, nor is there one for members of Generation Y, who are known as millennials. Some define millennials as people born from 1982 to 2004 [79]. Others define them as born between 1982 and 2000 [80], and some even use the years 1979 to 1994 [81].

The particular shared sociocultural experience that formed baby boomers was the postwar era, which was characterized by cultural radicalism and the rise of consumer society, whereas Generation X entered the workforce during an era of financial instability and recession [76,82]. In Finland, the deepest economic recession to date, which occurred during the early 1990s, also shaped the lives and careers of members of Generation X [83]. The most important event that has shaped millennial generation is rapid technological development. Millennials are digital natives who have used digital systems all their lives [84]. The Internet, mobile phones, and online social networks are also “millennials,” as they were evolved after the 1980s [85]. Digital technologies have high importance for millennials at work. For example, millennials perceive higher person–organization fit for a company with organizational policies that support employees’ social media use [86].

### 1.3. Millennials at Work

Many studies have suggested that generations are distinctive in terms of how they behave in work life [77,81,87]. However, not all studies confirm these stereotypes [76,88]. Thus, the picture of millennials remains unclear. For example, millennials do not value traditional wage employment compared to previous generations [89]. However, millennials also report a high degree of preference for materialistic rewards [90] and seek meaningful and engaging work [91].

Furthermore, researchers have found that millennials are more positive and collaborative than previous generations. In addition, they are more willing to change jobs in search of increased leisure or a more challenging and satisfying work environment [92]. Furthermore, they have higher levels of overall company and job satisfaction, career development and advancement compared to baby boomers and members of Generation X [93]. Overall, millennials value organizational attributes such as humane and informal organization cultures that they can influence [94].

In some studies, millennials did not differ strongly from other generations. For instance, in a study of young people’s work orientation in Finland over the past three decades, the value to employment showed signs of permanence and continuity among millennials. Thus, the results did not support the suggestion that young people’s work orientation is weakening [88]. In addition, in a U.S. study, the effects of generational membership on workplace behavior were not as strong as commonly held stereotypes suggest. According to this study, baby boomers exhibit fewer job mobility behaviors and more instances of compliance related behaviors compared to both members of Generation X and millennials. In addition, Generation Xers were less likely to work overtime compared to baby boomers and millennials. However, the effect sizes for these relationships were small [76].

Each generation has its own motivations, expectations, and career goals, which individuals bring to the workplace. This constitutes a challenge for managers in terms of understanding and balancing such differences, as well as avoiding intergenerational conflicts [95].

### 1.4. The Present Research

Although social media use has increased in organizations, there remains a gap in the current literature regarding how various generations use social media and what their motivations are for such use. Millennials are generally considered technologically savvy. However, is there a real difference in their technology use, and in particular social media use for work purposes, and do they actually cope better with technology compared to their older colleagues? This article is theoretically based on self-determination theory. We set the following two research questions (RQs) for our mixed-methods study:

RQ 1: How do millennials describe their motivations and social media use methods at work in qualitative expert organization employee interviews?

RQ 2: How are different motivations for social media use at work associated with technostress, burnout, and psychological distress in expert organizations and among Finnish employees?

Our findings supply important knowledge about social media use motivations and the association of well-being with employees of various ages. Our methodological triangulation and the two research goals provide diverse information on social media use at work among different generational groups and the connection to employee well-being.

## 2. Method

### 2.1. Participants

We based our study on three data samples collected for a research project investigating social media use at work and well-being at work. We used a sequential exploratory strategy for this mixed-method study as we first analyzed qualitative data followed by an analysis of the quantitative data in the second phase further building on the qualitative analysis [96]. We selected a mixed-method approach because the data sets complement each other and provide a multidimensional view on social media use at work. The qualitative data facilitates the articulation of explanations for social media use. In addition, we analyzed this use quantitatively and extended our scope to explore well-being implications for users of various ages first within the professional organizations and then nationally (see Table 1).

We conducted focus group interviews (*N* = 52) in five Finnish expert organizations (finance, telecommunications, personnel services, publishing, and retail occupational fields) during February and March 2018. In this article, we define expert organization as organization that employs highly skilled and educated employees, i.e., white collar knowledge workers and provides services or products related to knowledge or specific sophisticated solutions. The focus group interviews addressed 14 open-ended questions about social media use at work and well-being at work. The average duration of each interview was approximately 46 minutes. We recorded and transcribed all interviews. The respondents’ mean age was 32 years, with a range of 25–38 years, and 69% of the interviewees were women. All interviewed employees were qualified professionals or supervisors.

Employees of five Finnish expert organizations (finance, telecommunications, personnel services, publishing, and retail occupational fields) completed the *Social Media at Work in Expert Organizations Survey* during November and December 2018. The ages of participants (*N* = 563) ranged from 21 to 67 years (*M* = 40.7, *SD* = 10.9); 67.7% of the respondents were female, 31.6% were male, and 0.7% were other. The survey response rate ranged from 3.2% to 34.2% (*M* = 17.7, *SD* = 11.9).

Finnish employees, both white collar and blue collar, from various occupational fields completed the nationally representative *Social Media at Work in Finland Survey* in March and April 2019 (*N =* 1817; 46.84% female; *M*, age = 41.75; *SD*, age = 12.19). We collected the data in collaboration with Norstat, whose panel was used. We applied sampling weights to correct minor biases related to gender and age in the analyses.

### 2.2. Research Design and Procedure

We collected focus group interviews at five expert organizations in various occupational fields in Finland to gain an in-depth understanding of millennials’ social media use motivations at these organizations. A focus group interview is a facilitated group discussion focused on a certain research topic to gather a wide-ranging set of experiences and perspectives [97]. We recruited the selected companies via telephone and e-mail, and the companies participated free of charge. All focus group interviews were conducted on the respective companies’ premises. The company contact person, who was frequently Human Resources Manager or equivalent, recruited the research participants with an invitation that introduced the research and the research group.

We held two focus group interviews at each company over the course of a single day, one following the other. An average of five interviewees participated in each focus group, and these ranged from four to six participants due to no-shows. The ideal focus group size varies from six to eight participants, but the group size can vary from five to 10 participants [97]. Nevertheless, focus groups can be successful even with three participants [98]. No-shows are unavoidable, and approximately 20% participant loss is acceptable [99].

Participants completed the surveys online using either computers or mobile devices. The research group designed the expert organization survey using the LimeSurvey program [100], which was administrated by the research group on the university server. Norstat collected the national survey by utilizing their panel working aged members [101]. The surveys were aimed at discovering the social media use and factors related to employee well-being. The participants were informed of the study’s aims and their right to withdraw from the study at any point during data collection. Participation in the study was voluntary. The Academic Ethics Committee of Tampere region granted approved the research (90/2018).

Collecting responses to an identical survey in expert organizations and at the national level allowed us to establish a more extensive view on social media use and its well-being implications. The data sets used allowed us to compare and determine whether the results from expert organizations are replicated in the general workforce population. Our study design offers a novel perspective on the connections between social media use and well-being and enables us to discover insights from professionals that can be generalized to the Finnish workforce.

### 2.3. Measures of Quantitative Data

**Motivations for Social Media Use at Work.** We asked the respondents to list 11 motivations for their social media use at work (see Appendix A). The respondents could select all applicable options. The five main motivations were used to match the results based on qualitative analysis. Our analysis categories were as follows: information seeking, communication (communication with the work community), content production, content sharing, and networking. All these measures were dummy variables. All measures are summarized in Table 2.

**Social Media Use.** We measured daily social media use utilizing items in which respondents were asked to indicate how frequently they used 15 social media platforms. The list included the most popular platforms such as Facebook, Twitter, and Instagram. See Appendix B for the full list and answer options. We report descriptive findings about these variables in the text. The models utilize daily social media use variable as a control variable. This measure was created by counting the total amount of different social media platforms used on daily basis. The scale ranged from 0 to 15.

**Technostress.** We measured technostress in the expert organization sample using four items adapted from [102] technostress scale to measure the invasive and addictive sides of social media use. The adapted items were “I feel tense and anxious when I work with social media,” “I feel I use social media excessively in my life,” “I seem to have an inner compulsion to use social media in all places and at all times,” and, “It is difficult for me to relax after a day’s work using social media.” The scale for each item ranged from 0 (*never*) to 6 (*always*). The final scale had a good inter-item reliability of α = 0.81. The scale ranged from 0 to 24. In the nationwide sample, we measured technostress using the six items related techno-overload and techno-invasion by Ragu-Nathan et al. (2008) [20]. We adapted the items to social media. Example items include “I am forced to do more work than I can handle due to social media,” “I must always be available due to social media,” and “I feel my personal life is being invaded by social media.” For all items, the scale ranged from 1 (*disagrees completely*) to 7 (*agrees completely*). The scale showed a good inter-item reliability of α = 0.89 The scale ranged from 6 to 42.

**Burnout.** We measured burnout using the Maslach Burnout Inventory General Survey (MBI-GS) [103]. The original version of MBI-GS was validated with various occupational groups across nations [104]. The 16 items of MBI-GS scale were divided into three sub-scales of exhaustion, cynicism, and professional efficacy. They include questions such as “I feel tired when I get up in the morning and have to face another day on the job.” The answer scale ranged from 0 (*never*) to 6 (*every day*).” The scale showed good inter-item reliability of α = 0.89 in the expert organization sample, and α = 0.88 in national sample). The scale ranged from 0 to 96.

**Psychological Distress.** We measured psychological distress using the 12-item General Health Questionnaire (GHQ-12) [105]. The questions included items such as “Have you recently been able to enjoy your normal day-to-day activities *(More so than usual–Same as usual–Less so than usual–Much less than usual)*?” and “Have you recently been thinking of yourself as a worthless person *(Not at all–No more than usual–Rather more than usual–Much more than usual)*?” The scale showed good to excellent inter-item reliability of α = 0.89 in the expert organization sample, and α = 0.92 in national sample. The scale ranged from 12 to 48.

**Background Variables.** We used remote work, weekly working hours, education attainment, living arrangements, age, and gender. The descriptive statistics for all samples are reported in Table 2. For the nationally representative data set, probability weights were used when calculating the descriptive estimates.

### 2.4. Analysis Techniques

The first part of our study (RQ 1) was qualitative. We divided the overarching motivations of social media use into intrinsic and extrinsic use motivations based on SDT [66], which we used as a theoretical framework for the analysis. Although the content analysis was initially based on SDT, our scope was developed during the analysis process more towards data driven analysis to also discover methods of social media use (active versus passive) and benefits and strains related to usage. The interview transcripts were coded deductively by two researchers and cross-checked to confirm the reliability. Coding results were discussed together in detail and concluded to mutual agreement on coding. The qualitative analysis provided a starting point for the quantitative analyses (RQs 2 and 3).

To analyze how different motivations for social media use at work associate with occupational well-being (RQ 2), we conducted linear regression models predicting technostress, burnout, and psychological distress. For each model, our independent variables were the motivations of information seeking, communication with work community, information sharing, networking, and content production. In addition to these variables, we controlled for remote work, weekly working hours, education attainment, living arrangements, age, gender, and the total amount of different social media platforms used on a daily basis. We conducted all models separately for millennials and other older employees. Assumptions of regression analysis were checked, and we found no issues with multicollinearity. Due to the heteroscedasticity of residuals, we report robust (Huber-White) standard errors. For each model, we report unstandardized regression coefficients, standard errors, statistical significance of the estimates (*p* value), coefficients of determination (R^2^). We utilized sampling weights in all models.

## 3. Results

### 3.1. Millennials’ Social Media Use

*The intrinsic use motivations* were connected to pure personal choice and interest in using social media. Social media was used for professional development purposes and relationship building with both colleagues and other networks. Employees with *intrinsic social media use motivation* were genuinely interested in following and contributing to social media forums for the latest news and knowledge. One of our interviewees referred to their own choice in using social media without feeling any pressure:

“*You might be reading, sharing, or familiarizing yourself with some content that is related to your work through social media (e.g., LinkedIn), but there is no pressure or conflict, but it’s your own choice*”.

(Finance, Group 2).

*The extrinsic social media use motivation* was connected to work roles and organizations’ work culture. For some interviewees, social media use was self-evident and part of their work role (e.g., in communication, marketing, and HR positions). Interviewees also used social media for personal and employer branding. Some stated that social media use is nowadays an evident work tool, especially in certain industries, as one must keep up with the latest trends and follow the actions of clients and competitors. Others, on the other hand, used it mainly due to social pressure from the company, work community, or stakeholders. Social media platforms were also used for organizing work; thus, presence to some extent is required. Extrinsically motivated users used terms such as social selling with negative connotations: “*It creates certain pressure that you need to follow and know what is going on so that you don’t miss anything essential*” (Retail, Group 1). In this quote, the interviewee expressed a fear of missing out on important information, which refers to the fact that social media is such an important tool in their field of work and that there is pressure for using it. 

The interviewees had both *active and passive ways of using social media*. We defined active use as use that is visibly for other users and that can include active social interaction with others. Active users used social media for sharing work-related content and, for example, articles or news with their own insights and not merely for reposting, both internally and externally. Moreover, these users were actively starting and participating in discussions, thus aiming also to influence the followers. Instead, passive use, which we defined as not visible to others and having restricted social interaction, was limited to following the social media news feeds, retrieving and storing information, and occasionally reacting by liking and reposting other users’ posts. Passive users rarely shared their own content, as one of our interviewees described,

“*I just share everything, routinely—for example, if there are projects that I’m involved in and there are some positive news, I just share those links. That is basically how I use social media overall. I do not share anything personal*”.

(Publishing, Group 1).

We divided the consequences of social media use into *benefits and strains*. The consequences were not directly related to use motivation or if the use was active or passive: Also, those with strong intrinsic motivation reported strains, and those who were using social media more passively with more extrinsic motivation reported clear benefits of the use. The reported benefits of social media use included the rapidity of social media in terms of messaging and distributing knowledge, in addition to information accessibility, collaboration with the work community across the company, and internal and external networking. Interviewees reported that they can regulate the use themselves, which was seen very positively. In the following quote, social media is also seen as a tool for building trust in the work community:

“*So, it’s easier to stay connected and create a sense of community and build trust–that way, everything improves*”.

(Telecommunications, Group 1).

The perceived social pressure for using social media was stated as straining, for example, when clients are contacting any time of the day. In addition, time management issues were prevalent with endless social media feeds and the possibility for constant connectivity. Unclear social media rules and practices were also reported as a straining element. Furthermore, negative content and comments that people come across or are tagged into in social media were straining the respondents, especially if organizations did not have clear guidelines for such situations. The interviewees also reported psychological and physical strains such as fears (e.g., missing out, skills) and musculoskeletal disorders (e.g., neck pain). Social media may be connected with mental strain and, for example, insomnia. For instance, one of our interviewees reported bad feelings caused by social media: “*Of course, one straining element is that sometimes clients state their dissatisfaction in social media and you are kind of dealing with the same things at work, so it might feel bad*” (Publishing, Group 1).

Table 3 summarizes the previously presented millennials’ social media use motivations, types of use, and outcomes. Overall, millennials’ social media use can be crystalized into five user architypes, which can also be mapped to contribute to fulfilling basic psychological needs, information seekers (autonomy and competence), communicators (relatedness), content sharers (autonomy), content producers (competence) and networkers (relatedness) that were also used as a basis for quantitative analysis.

### 3.2. Associations between Social Media Use and Well-Being

Our analysis of expert organization workers and national workers showed that social media use at work was very common in Finland. Of the expert organization millennial respondents, 99.47% (560/563) used social media at work. In the national sample, 80.07% of millennials and 76.99% older employees had used social media at work. The difference between millennials and former generations was not statistically significant. Millennials reported higher technostress in both samples (*p* < 0.001). They also reported higher burnout in an expert organization sample (*p* = 0.049) and national sample (*p* < 0.001). Millennials also reported higher psychological distress in a national sample (*p* = 0.002).

We build our main analysis on user motivations of social media use that were grounded in qualitative research. The results based on expert organization workers showed that motivations of social media use were not associated with technostress, burnout, and psychological distress at the level of *p* < 0.05 among millennials (see Table 4). However, millennials who produced social media content reported lower technostress (*b* = −1.16, *p* = 0.093) and burnout (*b* = −4.96, *p* = 0.083). Information seekers among former generations reported lower burnout (*b* = −4.10, *p* = 0.047). Some of the control variables were significant in the millennial models. Millennial women reported more technostress than men, and daily social media use was associated with technostress. Those living alone had higher burnout scores. Among former generations, technostress was higher for females, younger workers, and those working less than 35 h per week. Daily social media use was associated with higher psychological distress.

We also found some differences among the national Finnish workers sample (see Table 5). Among millennials, networking (*b* = 2.24, *p* = 0.001) and content production (*b* = 2.91, *p* < 0.001) were associated with higher technostress and information seeking with lower technostress (*b* = −1.28, *p* = 0.032), and communication with the work community was associated with lower burnout scores (*b* = −4.13, *p* = 0.001) and psychological distress (*b* = −1.58, *p* = 0.001). Among older workers, information seeking (*b* = 1.64, *p* = 0.001), communication with the work community (*b* = 1.19, *p* = 0.011), and content sharing (*b* = 2.19, *p* = 0.002) were associated with higher technostress. Some of the control variables were also statistically significant within these models. Women reported higher psychological distress. Remote work had higher technostress in both millennials and others. Also, among former generations, the youngest respondents had higher technostress and burnout scores.

## 4. Discussion

### 4.1. Millennials as Social Media Users

This mixed method study used both focus group interviews and survey data to examine the motivations of social media use among millennials and former generations and their associations with technostress, burnout, and psychological distress. Our results contribute to the existing literature on social media use motivations and provide new knowledge and comprehensively analyzed and elaborated insights of the use motivations in the professional context. Our findings demonstrated that millennials have various intrinsic and extrinsic motivations for social media use at work. Intrinsic motivations are based on employees’ personal choice and their pure interest to follow the market, trends, and ongoing discussions in their own field. Employees are also personally motivated to use social media to enhance their skills and knowledge base and to build and maintain social relationships. These intrinsically motivated employees enjoy using social media for work purposes and see it as a benefit. Thus, the intrinsic motivation to use social media feeds the need to fulfill the basic psychological needs of autonomy, competence, and relatedness [67,68]. Our findings are aligned with the study by Demircioglu and Chen (2019) indicating that social media use is associated with employees need satisfaction and intrinsic use motivation [64].

By contrast, employees with extrinsic motivations for social media use at work are driven by external factors such as the fact that social media is an integral part of their work role or business or there is social pressure from the employer, colleagues, and stakeholders to use social media. The use may not always be pleasant, which may be since extrinsic motivations do not satisfy basic psychological needs [66]. This supports the findings of Panisoara and colleagues (2020) indicating that employees lack intrinsic motivation when they are not teaching online from their own will but are obliged to do so [18]. In the modern work life, employees increasingly use social media for personal and employer branding purposes [38,106]. Thus, the use is directed by external rewards such as maximized visibility and fame, enhanced career opportunities, and employer image, which are typical signs of extrinsic motivation [71]. Indeed, millennials have an urge for materialistic rewards in work life [90].

Furthermore, in our analysis, we divided social media use into active and passive use to elaborate the role of the user in more detail and to identify if the user activity is related to use motivations. Active use included internal communication and information sharing with colleagues, posting work-related content and sharing news, and articles and links with their own insights in internal and external social media platforms. Furthermore, active users participated in current discussions. Employees who stated that they use social media actively also normally enjoyed the use. Passive use was described as following social media feeds as well as reacting, liking, and reposting others’ content. Compared to active use, reposting was done without their own insight on the content. Employees also used social media for information seeking, retrieval, and storage. The main distinguishing point in passive use was that employees did not publish their own content actively. Motivations for social media use at work as such did not explain the activity of the use. Both intrinsically and extrinsically motivated employees can be actively using social media, but it can be argued that, in general, intrinsically motivated people have their basic psychological needs nurtured, experience positive feelings and well-being, and value social interaction e.g., [66,70,71], which can impact their activity on social media as well.

Millennials stated various benefits of using social media for work purposes. Information can be accessed quickly and limitlessly. Creating and maintaining networks and collaboration is easy and fast. Overall, millennials stated that rapidity was one of the most positive aspects of social media because messaging and sharing information with others are effortless. These findings support prior literature on the positive implications of social media use for work purposes e.g., [10,30,33]. Importantly, millennials indicated that they could regulate their social media use themselves; thus, autonomy played a key role in their use and positive view on it. Autonomy boosts intrinsic social media usage, which is also explored by Demircioglu and Chen (2019) [64]. The role of autonomy is critical to consider in organizations.

The results also showed that using social media for work was experienced as straining. Employees reported that they experience social pressure from the employer, colleagues, and stakeholders, which enhances their feeling of guilt if they are not active in social media, thus adding to the strain. Therefore, it is vital to consider that not all want to use social media, let alone become active users. Other mental and physical symptoms such as fears and sleeping problems contributed to employees’ strain. Indeed, social media use has been associated with, for example, sleeping problems in prior studies [43,44,107]. Millennials also longed for clear social media rules and guidance, which can help them solve difficult social media situations, hence reducing the burden. To support our finding, study by Cho and colleagues (2013) revealed that social media is very important for millennials and they experience higher person–organization fit for a company that promotes social media use in their organizational policies [86]. These are theoretically essential findings and important signals for practice.

### 4.2. Millennials: All Stressed and Strained?

With our cross-sectional survey data, we were able to examine the associations between social media use motivations and well-being at work among millennials and older employees, which has been lacking in the prior literature. Furthermore, we compared these with organizational data and the representative national data set. In line with prior studies regarding younger employees experiencing higher levels of IT-related strain [19,20], our findings demonstrated that millennials used social media more for work purposes and experienced higher technostress and burnout in both samples as well as higher psychological distress in the nationwide sample compared to former generations. Especially women and those millennials who used social media daily experienced higher technostress in the expert organizations and those working remotely in the Finnish workforce data. The findings are aligned with prior research indicating that intensified social media use [107], remote work [18] and female gender has been associated with heightened technostress [108]. In contrast, those expert organization older employees that worked shorter workdays reported more technostress and millennials living alone, reported higher burnout. Thus, situational factors play important role in decreasing employee well-being, which broadens the current knowledge of social media use at work.

The analysis of expert organization data revealed that motivations for social media use at work were not associated with technostress, burnout, or psychological distress, which provided new knowledge to the exiting theory and practice. Essentially, for millennials and older employees, various types of social media use can decrease technostress. Interestingly, millennials who produced social media content reported lower technostress and burnout (significant only with a 90% confidence level). For older employees, information seeking was associated with lower burnout. In contrast to our findings, previous studies have indicated that social media use for work purposes has been associated with increased burnout and technostress [21,41,43,109]. However, nearly a fifth of Finns have indicated that digitalization has decreased their work-related strain [22], which supports our findings. Our results imply that in the studied expert organizations, employees are fluent content producers indicating that their needs for competence have been fulfilled. Moreover, older employees rely on social media for information and solutions by satisfying their needs also for competence and autonomy. Therefore, these have buffering effect to the negative consequences of technostress. Indeed, former technological skills have been found to have an important role in accepting and utilizing new technologies [2,110]. Moreover, study by Molino and colleagues (2020) incited that personal resilience, possibilities for training and information enhanced the possibilities to accept new technologies into use and eventually fostered work engagement [8].

Among the Finnish workforce in general, remarkably, networking and content production were associated with higher technostress among millennials. Millennials are generally perceived as more positive and collaborative than previous generations [92]. Furthermore, the findings are interesting because, in the organizational data, content production was associated with reduced technostress. Thus, employees in expert organizations seem to be more experienced social media content producers compared to Finnish workers in general. Information seeking, in turn, was associated with lowered technostress and communicating with the work community with lowered burnout and psychological distress among Finnish millennials. Hence, seeking information and social interaction can serve as a buffer for the negative effects and can contribute to better well-being by aiding the psychological needs of competence, autonomy and relatedness. For instance, social support received in social media has been associated with positive outcomes such as enhanced work performance and work engagement and decreased work-related stress [111,112,113].

Our results also contributed to prior research on older employees’ social media usage and the related wellbeing implications. Among former generations, passive information seeking and content sharing and more active communication with the work community were associated with technostress. Therefore, older employees’ needs for autonomy, competence and relatedness may not be fully fulfilled in social media. According to Morris and Venkatesh (2000), older employees tend to base their technology use on social and process factors, thus contradicting our findings [4]. Additionally, remote work was linked to higher technostress among both groups. Thus, our findings are in line with earlier findings on the positive relationship of social media use, technostress and remote work e.g., [18,41,43].

To sum up, organizational results are not directly transferrable to describe the motivations of social media use and the related well-being implications among Finnish workforce and our study provides diverse findings to the current literature. For organizations, it is vital to acknowledge that employees have diverse motivations to use social media, which can depend on the age, situational factors and the organization they are working for. Based on our analysis, social media use motivations in expert organizations actually decrease the well-being burden of social media use to some extent. This is also true for millennial Finnish workforce, except those producing content have higher technostress. In contrast, some of the older employees’ social media use motivations are related to negative well-being consequences. Overall, however, our results indicate that millennials suffer more from the social media use although they may be more technologically equipped [24,84]. The underlying reason for this can be that their personal and work lives are currently overstimulated by social media, which can create fatigue, stress and strain for them [19,114].

## 5. Conclusions

Our research contributes to the theory and practice in several ways. It provides a multidimensional view on the motivations for social media use at work by different aged employees and the association to technostress, burnout, and psychological distress. With this study, we wanted to understand the motivations to use social media for work and the associated well-being implications by comparing millennials and former generations. The chosen sequential exploratory strategy [96] was sound and functional approach for this mixed-method study. The analyses were drawn from three different data sets consisting of qualitative and cross-sectional organizational survey data and a representative survey data of the Finnish workforce. We based motivation types for social media use at work on qualitative data and analyzed them cross-sectionally with two different data sets. Hence, the multiple data sets enabled us to provide a comprehensive view of the topic and provided important contribution to exiting literature on social media use motivations and related wellbeing implications for different aged employees, which is our key strength.

Various theories and frameworks such as TAM e.g., [56], gratifications theory e.g., [59], affordances e.g., [11] and utilitarian and hedonistic motivations e.g., [36] have been used to study social media usage in work context. However, not much research is done [63,64,65] with SDT developed by Ryan and Deci [68,69] regarding social media use motivations in work context. Therefore, our findings provide a considerable contribution to the theory by considering also the generational differences of motivation driven social media usage and the related wellbeing implications in addition to using SDT as theoretical framework. SDT suits well in social media use research because intrinsically motivated social media use stimulates the three basic psychological needs of individuals: autonomy, competence, and relatedness [68,69,115], which have been associated with enhanced well-being across nations [70].

Based on our analysis millennials have various intrinsic and extrinsic motivations for social media use at work. Intrinsic motivations include employees’ personal choice and their pure interest to follow the market and discussions in their own field. Extrinsic motivations are related mainly to organizations’ work culture and personal branding. Our survey results indicate that millennials experienced higher technostress and burnout. Moreover, the motivations for social media use at work differ among millennials and former generations and that the use motivations also varied in terms of their incising or decreasing impact on well-being.

Our results provide valuable insights for organizations to consider in their daily work practices; there is no single and right way to utilize social media for work purposes and individual differences must be acknowledged and respected. It is also important to recognize the mental burden related to social media usage and develop alleviating methods to support wellbeing and fight against the increasing contemporary problems of psychological distress, technostress and burnout at work. Furthermore, providing help and training to enforce employees’ psychological needs of autonomy, competence and relatedness is crucial. Overall, it can be implied that employees tend to utilize social media more if they see the personal advantage of the use rather than the employer demanding they use it. When employees feel they have the required competence to use social media, they can regulate the use of it themselves and have the opportunity to make meaningful connections with other people, they are intrinsically motivated and in a good state of mental health.

## Figures and Tables

**Table 1:** Study participants, data, and design.

Details	Organizational Focus Group Interviews	Organizational Survey	National Survey
*N*	52	563	1817
Sample population	Millennials of five expert organizations (different industries)	Various aged respondents of five expert organizations (different industries)	Various aged Finnish employees across different industries
Purpose	To define social media use motivations	To analyze the associations between social media use motivations and well-being	To examine whether the results from expert organizations are replicated in the general workforce population
Point of time collected	February and March 2018	November and December 2018	March and April 2019

**Table 2:** Descriptive statistics on expert organization sample (*N* = 563) and national sample (T1, *N* = 1817).

Continuous Variables	Scale	Expert Organizations				National Sample			
		Millennials		Former Generations		Millennials		Former Generations	
		*M*	*SD*	*M*	*SD*	*M*	*SD*	*M*	*SD*
Technostress	0–24/6–42	7.46	0.28	5.59	0.25	14.53	7.25	11.6	6.8
Burnout	0–96	34.63	15.36	31.99	16.08	39.27	15.26	36.29	16.98
Psychological distress	12–48	25.04	5.62	24.62	5.83	25.73	6.51	24.74	6.15
Daily social media use	0–15	4.46	1.78	3.25	1.64	3.82	2.01	2.38	1.68
Age	22–68	31.51	4.8	49.66	6.88	29.02	5.6	50.4	7.22
Categorical variables	Coding	*n*	%	*N*	%	*n*	%	*n*	%
Information seeking	No	49	19.8	70	22.2	444.78	59.46	693.93	64.91
	Yes	199	80.2	245	77.8	303.22	40.54	375.07	35.09
Communication	No	79	31.9	94	29.8	450.24	60.19	711.89	66.59
	Yes	169	68.2	221	70.2	297.76	39.81	357.11	33.41
Information sharing	No	115	46.4	135	42.9	546.33	73.04	823.97	77.08
	Yes	133	53.6	180	57.1	201.67	26.96	245.03	22.92
Networking	No	108	43.6	164	52.1	571.63	76.42	840.43	78.62
	Yes	140	56.5	151	47.9	176.37	23.58	228.57	21.38
Content production	No	165	66.5	208	66	588.32	78.65	914.86	85.58
	Yes	83	33.5	107	34	159.68	21.35	154.14	14.42
Remote work	No	85	34.3	76	24.1	537.48	71.86	735.62	68.81
	Yes	163	65.7	239	75.9	210.52	28.14	333.38	31.19
Working hours	<35 h	21	8.5	14	4.4	203.02	27.14	190.96	17.86
	35–40 h	182	73.4	199	63.2	408.12	54.56	626.1	58.57
	>40	45	18.2	102	32.4	136.85	18.3	251.94	23.57
Higher education	No	54	21.8	140	44.4	380.05	50.81	582.16	54.46
	Yes	194	78.2	175	55.6	367.95	49.19	486.84	45.54
Lives alone	No	183	73.8	271	86	520.38	69.57	832.44	77.87
	Yes	65	26.2	44	14	227.62	30.43	236.56	22.13
Gender	Male	72	29.4	106	33.8	397.48	53.14	548.71	51.33
	Female	173	70.6	208	66.2	350.52	46.86	520.29	48.67

**Table 3:** Millennials’ social media use at work.

**Intrinsic Use Motivation**	Personal choice Professional development Genuine interest to follow trends, market, discussions Relationship building	**Extrinsic Use Motivation**	Part of the role or business Social pressure (company, stakeholders) Personal and employer branding Organizing work
**Active Use**	Internal information sharing Posting work content Sharing news, articles, links Participating discussions	**Passive Use**	Following feeds Information retrieval and storage Reacting, liking, reposting Not publishing own content
**Benefits**	Information Rapidity Collaboration and networks Autonomy & self-regulation	**Strains**	Social pressure Time management Unclear rules Psychological and physical symptoms

**Table 4:** Linear regressions on associations of social media use motivations on psychological well-being among expert organization workers (*N* = 563).

Variables	Millennials									Former Generations								
	Technostress			Burnout			Psychological Distress			Technostress			Burnout			Psychological Distress		
	*b*	*SE*	*p*	*b*	*SE*	*p*	*b*	*SE*	*p*	*b*	*SE*	*p*	*b*	*SE*	*p*	*b*	*SE*	*p*
Information seeking	0.46	0.48	0.388	1.69	0.88	0.127	0.05	1.03	0.964	−0.07	0.37	0.856	**−4.1**	**1.44**	**0.047**	−1.17	1.11	0.352
Communication	0.61	0.99	0.569	−0.88	3.61	0.82	0.19	1.3	0.888	0.12	0.43	0.797	−0.48	3.08	0.885	0.78	0.68	0.317
Information sharing	0.93	0.54	0.158	−0.64	1.6	0.71	−0.1	1.09	0.932	0.42	0.77	0.614	−4.43	2.4	0.139	−0.64	1.23	0.629
Networking	−0.68	0.58	0.311	−0.71	2.15	0.759	−0.02	0.63	0.978	0.97	0.53	0.138	0.57	2.59	0.837	1.02	0.5	0.11
Content production	−1.16	0.53	0.093	−4.96	2.16	0.083	−1.01	0.87	0.309	0.45	0.6	0.5	−1.08	2.2	0.649	−0.7	0.85	0.457
Remote work (ref. no)	**1.6**	**0.27**	**0.004**	1.03	0.94	0.335	0.46	0.96	0.655	0.55	0.31	0.15	1.68	1.98	0.444	0.16	0.64	0.81
Working hours (ref. < 35 h)																		
35–40 h	0.36	1.04	0.746	6.85	4.47	0.2	2.29	1.29	0.151	**−2.77**	**0.87**	**0.033**	−1.68	5.02	0.755	−0.31	0.98	0.766
>40	−0.03	0.82	0.972	2.04	3.67	0.608	0.96	1.25	0.485	−1.36	0.67	0.112	1.77	6.06	0.785	1.27	1.05	0.295
Higher education (ref. no)	1.09	0.44	0.067	−0.15	2.97	0.961	−1.39	0.79	0.156	0.09	0.62	0.897	−0.81	0.36	0.087	−0.45	0.3	0.208
Lives alone (ref. no)	−0.11	0.3	0.725	**5.92**	**1.75**	**0.028**	0.65	0.79	0.456	0.21	0.9	0.83	3.37	3.71	0.416	1.76	1.08	0.179
Age	−0.05	0.02	0.106	−0.07	0.3	0.827	0.02	0.07	0.76	**−0.09**	**0.03**	**0.049**	−0.14	0.2	0.514	−0.07	0.05	0.28
Female	**2.21**	**0.66**	**0.028**	3.9	2.72	0.225	2.22	0.81	0.052	**1.95**	**0.49**	**0.016**	1.95	1.71	0.319	−0.11	0.29	0.712
Daily social media use	**0.41**	**0.12**	**0.026**	0.99	0.88	0.32	0.26	0.28	0.401	0.35	0.17	0.114	0.91	0.52	0.155	**0.41**	**0.14**	**0.042**
Constant	3.01	1.18	0.063	23.59	10.57	0.089	20.56	2.81	0.002	8.34	2.11	0.017	40.27	10.32	0.018	26.92	2.71	0.001
R^2^	0.15			0.1			0.07			0.19			0.07			0.06		

Note. Bold font indicates statistical significance (*p* < 0.05); R^2^ = R-squared.

**Table 5:** Linear regressions on associations of social media use motivations on psychological well-being among Finnish workers (*N* = 1817).

Variables	Millennials									Former Generations				
	Technostress			Burnout			Psychological Distress			Technostress			Burnout	
	*b*	*SE*	*p*	*b*	*SE*	*p*	*b*	*SE*	*p*	*b*	*SE*	*p*	*b*	*SE*
Information seeking	**−1.28**	**0.6**	**0.032**	−1.5	1.24	0.226	−0.63	0.52	0.222	**1.64**	**0.49**	**0.001**	1.36	1.2
Communication	−0.3	0.55	0.583	**−4.13**	**1.21**	**0.001**	**−1.58**	**0.5**	**0.001**	**1.19**	**0.47**	**0.011**	−0.15	1.17
Information sharing	0.46	0.74	0.536	2.18	1.44	0.13	0.81	0.62	0.192	**2.19**	**0.69**	**0.002**	0.42	1.56
Networking	**2.24**	**0.68**	**0.001**	1.01	1.53	0.51	0.49	0.66	0.456	0.71	0.62	0.257	−1.3	1.32
Content production	**2.91**	**0.81**	**<0.001**	−1.9	1.62	0.241	0.49	0.66	0.456	0.46	0.81	0.573	−1.64	1.82
Remote work (ref. no)	**1.46**	**0.63**	**0.021**	2.63	1.38	0.057	0.63	0.59	0.289	**1.39**	**0.5**	**0.006**	0.49	1.23
Working hours (ref. < 35 h)														
35–40 h	−0.65	0.64	0.309	−1.44	1.42	0.311	−0.1	0.59	0.868	0.22	0.5	0.653	−1.31	1.45
>40	0.18	0.82	0.824	−0.18	1.81	0.922	−0.36	0.77	0.638	0.7	0.65	0.283	−0.94	1.73
Higher education (ref. no)	−0.12	0.54	0.82	−1	1.22	0.414	−0.81	0.53	0.125	0.05	0.42	0.91	−1.21	1.11
Lives alone (ref. no)	0.19	0.57	0.742	0.97	1.3	0.455	0.77	0.56	0.171	0.02	0.45	0.96	1.27	1.3
Age	−0.09	0.05	0.105	−0.08	0.11	0.48	−0.05	0.05	0.386	**−0.07**	**0.03**	**0.014**	**−0.19**	**0.07**
Female	−0.6	0.53	0.257	0.37	1.17	0.751	**1.23**	**0.5**	**0.015**	0.41	0.39	0.287	−1.31	1.07
Daily social media use	**0.37**	**0.16**	**0.018**	−0.17	0.28	0.551	−0.23	0.13	0.091	**0.32**	**0.15**	**0.036**	0.31	0.32
Constant	15.77	2	<0.001	43.7	3.92	<0.001	26.68	2.02	<0.001	11.46	1.81	<0.001	48.22	4.57
R^2^	0.1			0.03			0.04			0.15			0.01	

Note. Bold font indicates statistical significance (*p* < 0.05). R^2^ = R-squared.

## Data Availability

The data presented in this study are available on request from the corresponding author. The data will be later on made publicly available at the Finnish Social Science Data Archive.

## Appendix A. For What Purposes Do You Use Social Media at Work? [Select All Applicable]

I do not use
Content following
Content production
Content sharing
Information seeking for work-related issues
Professional networking
Communication with the work community
To learn something about your colleagues
Keeping in touch with clients and other stakeholders
To enhance own career and visibility
To have a break at work
Communication with friends and family

## Appendix B. How Often Do You Use the Following Social Media Services for Work Purposes?

Facebook
Facebook Messenger
Workplace by Facebook
Twitter
LinkedIn
Instagram
Pinterest
WhatsApp
Snapchat
YouTube
Periscope
MS Teams
Yammer
Skype
SlideShare
Slack
Smarp
Trello Blogs (e.g., Tumblr)
Wiki-pages
Discussion forums (e.g., Suomi24, Reddit)
Some other social media service, which?
